# Association between sleep disorders and physical activity in middle-aged Americans: a cross-sectional study from NHANES

**DOI:** 10.1186/s12889-024-18665-w

**Published:** 2024-05-07

**Authors:** ZhiYing Fei, XiaoYing Zhu, QiDan Shan, FangYuan Wan, YingYing Tu, XiaoHeng Lv

**Affiliations:** Nursing Department, Sir Run Run Shaw Hospital, Zhejiang University School of Medicine, 3 Qingchundong Road, Hangzhou, Zhejiang Province 310016 People’s Republic of China

**Keywords:** Physical activity, Sleep disorders, NHANES, Middle-age American

## Abstract

**Background:**

Among the numerous studies on physical activity and sleep disorders, few have focused on physical activity and sleep disorders in middle-aged people who are particularly stressed. A restricted cubic web (RCS) technique was applied to determine whether physical activity and the self-rated prevalence of sleep disorders exhibit a dose-response relationship in middle-aged adults.

**Methods:**

This study analyzed 8880 middle-aged adults aged 40–65 years who participated in the National Health and Nutrition Examination Survey (NHANES) 2007–2018. Logistic regression was performed to estimate the odds ratio (OR) and 95% confidence interval (CI) between physical activity and sleep disorders in middle-aged adults. Thereafter, the dose-response connection was examined using RCS.

**Results:**

After adjusting for potential confounders, subjects with MET values in the first quartile (Q1) had odds ratios (OR) for sleep disturbance of 0.851 (95% CI = 0.745–0.973), 0.800 (95% CI = 0.698–0.917), and 0.780 (95% CI = 0.680–0.895) compared to subjects with MET values in the second, third, and fourth quartiles respectively. RCS regression showed a non-linear association between physical activity and sleep disorders in middle-aged adults (non-linearity *P* = 0.0382). Furthermore, the prevalence of sleep disorders in middle-aged adults decreased with increasing physical activity, reaching a minimum when weekly physical activity was around 166.27MET*h (OR = 0.885, 95% CI = 0.799–0.981).

**Conclusion:**

Our research demonstrates that physical activity was negatively associated with sleep disorders.

## Introduction

Sleep disorders are defined as the inability to get sufficient sleep in a recurring manner. Common sleep disorders include insomnia, sleep apnea, narcolepsy, and restless leg syndrome, which are among the most common clinical problems that disrupt sleep patterns [1]. The prevalence of sleep disorders has increased over the past few decades and has reached high levels, with a recent study showing a prevalence of 27.1% of U.S. adults [2]. Among all age groups, middle-aged people are more prone to develop sleep disorders due to significant mental stress and aging bodies. Sleep disorders are associated with various negative health consequences, such as reduced quality of life and productivity, and increased risk of medical and psychiatric problems [3]. Moreover, sleep disorders are risk factors for various illnesses, including cardiovascular incidents [4], hypertension [5], and type 2 diabetes [6], particularly in the elderly [7] and populations who are under psychological stress. In clinical practice, a number of simple lifestyle adjustments can be applied to treat sleep disorders.

Physical activity constitutes an essential lifestyle change to improve the quality of sleep for middle-aged and elderly people [8]. In recent years, physical activity has been linked to improvements in multiple ailments. For instance, physical activity has been proven to be beneficial for mild cognitive impairment (MCI) and dementia [9]. In addition, functional recovery in patients with bidirectional depression [10], neuroendocrine abnormalities in Parkinson’s disease [11], restless leg syndrome (RLS) [12], have all demonstrated improvement. Furthermore, research suggests that increased physical activity and fitness may be a potential prevention and/or treatment pathway to reduce sleep disorders [13, 14]. However, all of these studies only categorize sports in a general way and lack quantifiable criteria. Therefore, this study aimed to investigate the dose-response association between sleep disorders and physical activity in a large, nationally representative sample of middle-aged adults.

Restricted cubic spline (RCS) functions are powerful tools to characterize a dose-response association between a continuous exposure and an outcome [15]. The methodology of applying RCS to evaluate dose-response associations has been widely reported [16, 17]. Therefore, RCS and logistics regression were used to study the relationship between physical activity and sleep disorders in middle-aged people, providing a reference for the prevention of sleep disorders.

## Materials and methods

### Population and data sources

**Fig. 1:**
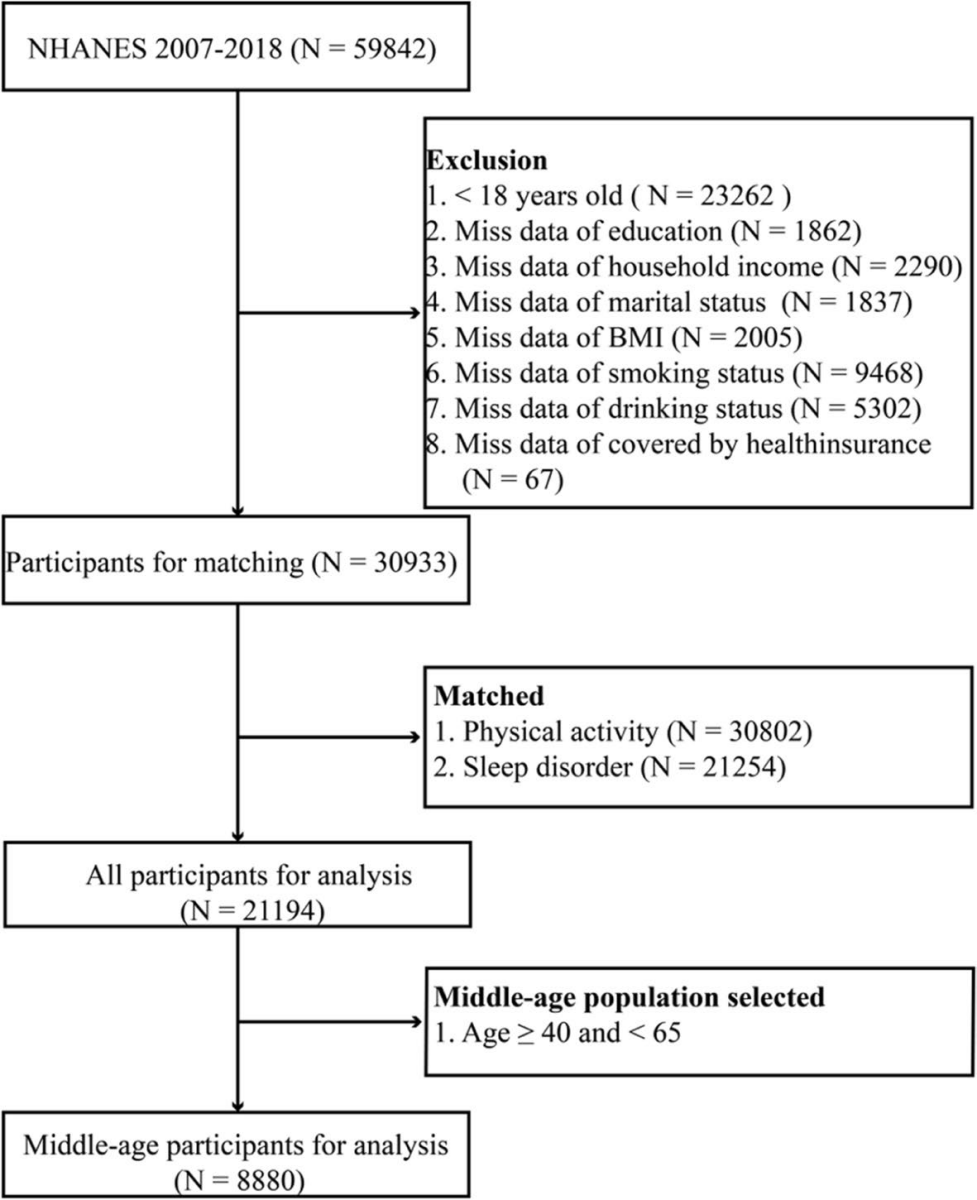
Flowchart of the population included in our final analysis

The National Health and Nutrition Examination Survey (NHANES) (https://wwwn.cdc.gov/nchs/nhanes/Default.aspx) is a cross-sectional research project designed and implemented by the Centers for Disease Control and Prevention (CDC) since 1999 that collects data on health examinations of the outpatient population in the United States [18]. The study was approved by the National Center for Health Statistics (NCHS) Institutional Review Board for Research Ethics, and the database provides a nationally representative population in the U.S. NHANES is a multi-level, complex, large-scale, population-wide probability sampling survey. In this study, data from six consecutive NHANES biennial cycles (2007–2018) were analyzed, which included a total of 59 842 participants. Among these participants, 23 242 participants were excluded as they were under the age of 18, 1862 participants were excluded due to having incomplete education information, 2 290 participants were left out owing to unavailable household income data, 1 837 participants were excluded for missing values for marital status, and 2005 participants were not included due to missing values for BMI. Furthermore, 9468 participants with missing values for smoking status, 5302 missing values for alcohol consumption, and 67 missing values for Social Security were excluded. The demographic, social, sleep disturbance, and physical activity data were excluded. Finally, 8880 participants aged 40–65 years were enrolled in the study (Fig. 1).

### Outcome variables

Sleep disorders were assessed using the Sleep Disorders Questionnaire and participants were asked whether they had been told by a doctor or other health professional that they had a sleep problem [19]. Subsequently, participants were grouped based on their responses (yes or no), and those who chose not to respond or did not know were considered missing data.

### Exposed variables

Each NHANES participant completed a physical activity questionnaire based on the Global Physical Activity Questionnaire (GPAQ) [20]. The questionnaire asked about the type, frequency (days of the week), and duration of physical activity (at least 10 min of physical activity in the previous 30 days, typically in one day), including leisure activities (moderate and vigorous intensity activities were counted separately), walking or riding a bicycle for exercise, and work (moderate and vigorous intensity activities were counted separately). Vigorous activity was defined as activity that causes a large increase in respiration or heart rate, and moderate activity was defined as activity that causes a small increase in respiration or heart rate. Labor-related physical activity included both paid or unpaid work, housework, and courtyard work. Recreational physical activity referred to sports, fitness, and recreational activities. Information on all walking and cycling activities was collected in the active transport domain.

The term “MET” was used to indicate the amount of energy required to perform a particular task, where 1 MET = 3.5 ml O_2_ kg^−1^ min^−1^. The METs for vigorous work-related activity, moderate work-related activity walking or cycling, vigorous spare time physical activity, and moderate amateur physical activity were 8.0, 4.0, 8.0, and 4.0, respectively. The amount of physical activity was measured in MET-min. Subsequently, the sum of the days, the average duration, and the projected MET were multiplied to determine the total physical activity.

### Accompanying factors

Sociodemographic, behavioral, and health traits that were presumptively thought to be possible confounders were analyzed as relevant variables.

The socio-demographic variables included the age group, gender (male and female), race (Mexican-American, other Hispanic, white, non-black and other races, including multiracial), education level (< high school, high school diploma, some college and college degree or more), family income (< 20 000, 20 000–44 999, 45 000–74 999 and > 75 000), and marital status (married, widowed, divorced, separated, never married, living with partner).

The behavioral characteristics included smoking (never, ex-smokers and currently smoking) and alcohol usage (not drinking and drinking).

Health factors: body mass index BMI, social security (absent or present).

### Statistical methods

Data were expressed as mean and standard deviation for continuous variables and as percentages for categorical variables. Categorical and continuous variables were tested using the t-test and chi-square test, respectively. In addition, logistic regression was performed to explore the relationship between physical activity and sleep disturbance, adjusting for sociodemographic, behavioral, and other confounders. Two adjusted models were established to test the reliability of the results. Model 1 was adjusted for age, sex, and race/ethnicity, while Model 2 was further adjusted for education level, marital status, annual household income, and social and behavioral confounders.

Using the 25th, 50th, and 75th exercise percentiles as fixed nodes, the RCS was performed to test the dose-response association between exercise and sleep disorders. A non-parametric test of the nonlinear relationship between the amount of exercise and sleep disturbance was performed using the RCS model. The RCS models were adjusted for age, sex, race, education level, household income, marital status, social security status, smoking, alcohol consumption, and body mass index. Data were transposed and analyzed using the R language (4.1.2) and *P* < 0.05 was considered statistically significant.

## Results

### Baseline information

The distribution of the major factors evaluated in this study among middle-aged persons with and without sleep disturbances is shown in Table 1. Of the 8880 people included in this analysis, 3534 (39.8%) had a sleep disorder, including 1448 (41.0%) women and 2086 (59.0%) men. The mean MET values of subjects with sleep disorders were significantly lower than those of subjects without sleep disorders (*P* < 0.001). In addition, the presence of sleep disturbances was significantly associated with sex (*P* < 0.001), age (*P* < 0.001), race (*P* < 0.001), education (*P* < 0.001), household income (*P* < 0.001), marital status (*P* < 0.001), and MET (*P* < 0.001), body mass index (*P* < 0.001), smoking status (*P* = 0.002), alcohol consumption status (*P* < 0.001), and health insurance status (*P* < 0.001). In conclusion, participants with sleep disturbances were more likely to be female, older, non-Hispanic white, students, married, nonsmokers, have a low income, not consume alcohol, and have a higher body mass index.

**Table 1:** Baseline characteristics of participants aged 40–65 years with and without any history of sleep disorder: NHANES survey 2007–2018

Group	Sub-group	Non-sleep disorder	Sleep disorder	*P*
n		5,346	3,534	
Gender^a^	Men	2765 (51.7)	1448 (41.0)	< 0.001
	Women	2581 (48.3)	2086 (59.0)	
Age^b^(Year)		51.82 (7.40)	52.77 (7.05)	< 0.001
Race^a^	Mexican American	982 (18.4)	398 (11.3)	< 0.001
	Other Hispanic	616 (11.5)	336 ( 9.5)	
	Non-Hispanic White	2012 (37.6)	1666 (47.1)	
	Non-Hispanic Black	1210 (22.6)	827 (23.4)	
	Other Race - Including Multi-Racial	526 ( 9.8)	307 ( 8.7)	
Education_level^a^	< High school	1418 (26.5)	737 (20.9)	< 0.001
	High school graduate	1177 (22.0)	830 (23.5)	
	Some college	1422 (26.6)	1142 (32.3)	
	College graduate or above	1329 (24.9)	825 (23.3)	
Household_income^a^($)	< 20,000	903 (16.9)	856 (24.2)	< 0.001
	20,000–44,999	1698 (31.8)	1033 (29.2)	
	45,000–74,999	1047 (19.6)	659 (18.6)	
	> 75,000	1698 (31.8)	986 (27.9)	
Marital_status^a^	Married	3340 (62.5)	1865 (52.8)	< 0.001
	Widowed	184 ( 3.4)	175 ( 5.0)	
	Divorced	768 (14.4)	695 (19.7)	
	Separated	225 ( 4.2)	180 ( 5.1)	
	Never married	517 ( 9.7)	424 (12.0)	
	Living with partner	312 ( 5.8)	195 ( 5.5)	
BMI^b^(kg/m^2^)		29.23 (6.41)	31.73 (8.26)	< 0.001
Smoking^a^	Current smoker	1137 (21.3)	956 (27.1)	< 0.001
	Ex-smoker	1222 (22.9)	967 (27.4)	
	Never smoker	2987 (55.9)	1611 (45.6)	
Drinking^a^ (%)	Drinker	1483 (27.7)	1091 (30.9)	0.002
	Non-drinker	3863 (72.3)	2443 (69.1)	
Covered_by_health_insurance^a^	Yes	3755 (70.2)	2958 (83.7)	< 0.001
	No	1591 (29.8)	576 (16.3)	
Physical activity^b^ (MET/week)		59.27 (100.87)	49.83 (90.50)	< 0.001

^a^Categorical variable

^b^Continuous variable (mean ± SD)

Table 2 presents the odds ratios (OR) and 95% confidence intervals (CI) for the quartiles of MET sleep disorders. Participants in the first quartile (Q1) constituted the reference group (OR = 1.00). Univariate analysis revealed that compared with Q1 MET, the OR of sleep disturbance in the Q2, Q3, and Q4 MET groups were 0.854 (95% CI = 0.759–0.961), 0.784 (95% CI = 0.696–0.883), and 0.695 (95% CI = 0.616–0.783), respectively (all *P* < 0.05).

**Table 2:** Odds ratios (95%CI) of sleep disorder among different physical activity levels

Group	Crude	Model 1	Model 2
Physical activity			
Q1 (0 MET/week)	Ref (1)	Ref (1)	Ref (1)
Q2 (< 16 MET/week)	**0.854(0.759, 0.961)**	**0.837(0.742, 0.944)**	**0.851(0.745, 0.973)**
Q3 (16 ~ 60 MET/week)	**0.784(0.696, 0.883)**	**0.792(0.701, 0.894)**	**0.800(0.698, 0.917)**
Q4 (> 60 MRT/week)	**0.695(0.616,0.783)**	**0.758(0.670, 0.858)**	**0.780(0.680, 0.895)**

Model 1: Age, gender, and race/ethnicity were adjusted

Model 2: Adjustments in Model 1 plus educational level, marital status, annual family income

BMI, smoking status, and drinking status

*Ref* Reference

After adjusting for confounding factors such as age, sex, race, education level, household income, marital status, social security, smoking, alcohol consumption, and body mass index, the OR for sleep disturbances were 0.837 (95% CI = 0.742–0.944), 0.792 (95% CI = 0.701–0.894), 0.758 (95% CI = 0.670–0.858) and 0.851 (95% CI = 0.745–0.973), 0.800 (95% CI = 0.698–0.917) 、0.780 (95% CI = 0.680–0.895) (all *P* < 0.05) in subjects with MET Q2, Q3, and Q4 compared with those with MET Q1 values.

**Fig. 2:**
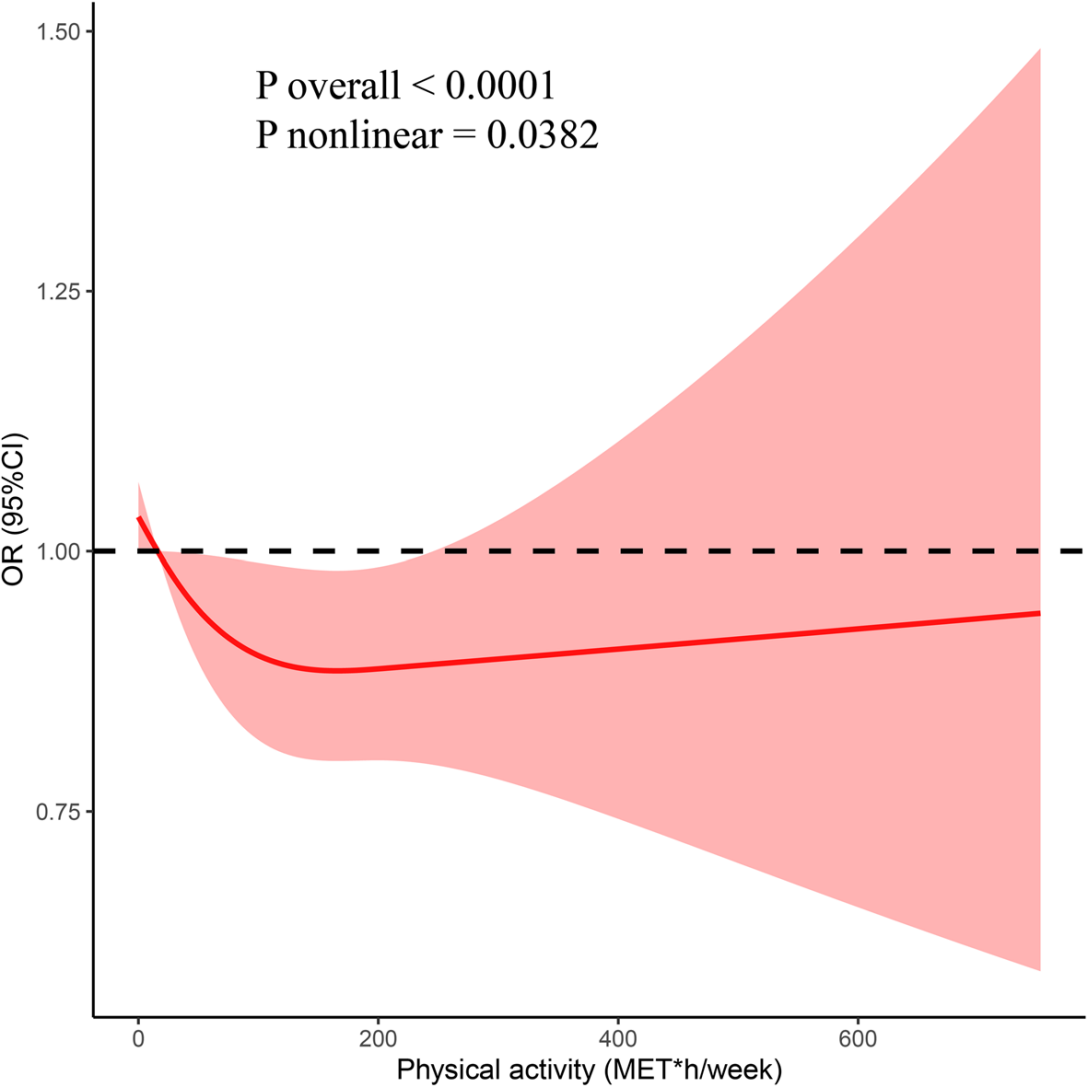
Adjusted cubic spline models showing the association between physical activity and the prevalence of sleep disorders in all participants

Furthermore, the RCS model showed a non-linear relationship between physical activity (PA) and the incidence of sleep disturbances in all subjects (non-linear *P* = 0.0382) (Fig. 2). The amount of physical activity ranged from 0 to 166.27 MET*h per week. As physical activity increased, the OR for sleep disorder gradually decreased, reaching its lowest point at 166.27 MET*h per week (OR = 0.885, 95%CI = 0.799–0.981). These results implied that after reaching a certain level of physical activity, the prevalence of sleep problems showed no further significant decline as physical activity was increased. In contrast, the prevalence of sleep disorders increased slightly with increasing physical activity once physical activity reached a certain level. The dose-response relationships between physical activity and sleep disturbance were consistent with a logistic model in which no significant results were presented.

## Discussion

This study is the first to apply restricted cubic spline (RCS) analysis and logistic regression to delineate the relationship between physical activity and sleep among middle-aged adults, offering precise activity thresholds to guide the formulation of targeted exercise programs for this demographic. A prospective study has shown that the use of low-to-moderate intensity exercise is effective in maintaining adequate sleep in middle-aged and older adults, which aligns with our findings. However, that study grouped exercises into three broad categories and focused on a combined cohort of middle-aged and older adults. Similarly, multiple recent studies did not distinguish between middle-aged and older adults [21, 22]. Yet, evidence suggests that high workloads and elevated stress levels not only increase sleepiness and extend work hours but also impair sleep quality and alter diurnal cortisol secretion patterns [23, 24]. Considering that middle-aged individuals are more frequently subjected to high workloads compared to their older counterparts, the middle-aged population should be investigated separately. The NHANES database was used to explore the link between physical activity and sleep disorders in middle-aged people, providing a nationally representative sample. In this study, 3 534 (39.8%) of the 8 880 individuals enrolled in the National Health Interview Survey (NHANES) for the period 2007–2018 were found to be sleep-deprived. In general, physical activity was lower in patients with sleep disorders than in those without any sleep disorder. Logistic regression analyses revealed that physical activity was an independent protective factor for sleep disorders. In addition, an RCS analysis was performed after adjusting for confounding factors, demonstrating that physical activity was negatively correlated with sleep disturbance (non-linear association) in all participants. The OR of sleep disorders decreased with increasing physical activity, then reached a turning point; further increases in physical activity resulted in a slight increase in OR of sleep disorders.

Other studies have investigated the link between physical activity and sleep disturbance. One study found a significant negative association between strenuous exercise and sleep disorders [25]. The circulating concentrations of ACTH and cortisol increase with exercise intensity [26]. Another key function of the neuroendocrine system is the regulation of the circadian rhythm, which is regulated by the central circadian clock and the suprachiasmatic nucleus (SCN) [27]. The key output pathway of the SCN is its projection to the pineal gland, which produces melatonin. These input and output pathways are therefore reciprocal. The neurohormone melatonin acts as a transmitter of signals, coordinating and stabilizing circadian rhythms and regulating hormone secretion, body temperature, cognition, and mood [28]. Melatonin secretion is also affected by exercise intensity and time. High-intensity exercise and morning exercise increase melatonin levels more than moderate-intensity exercise and afternoon exercise, resulting in a greater increase in melatonin at night [29]. The second exercise increased PTPRD (protein tyrosine phosphatase receptor delta-type) protein levels, decreased tyrosine hydroxylase (TH) protein levels, and improved sleep parameters in both cycles of restless leg syndrome. The results of this study showed an improvement in sleep parameters in both cycles of restless leg syndrome  [12, 30]. However, one study showed that excessively strenuous physical activity disrupts redox homeostasis against oxidative stress [31], which disrupts the antioxidant status of the body and thus affects sleep quality. This is consistent with the results of the RCS model in the current study, demonstrating that the incidence of sleep disorders increases slightly as physical activity reaches a certain level.

The major strength of this study was the use of a sizable sample from a nationally representative. The correlation between physical activity and sleep disorders in middle-aged adults was estimated using logistic regression and RCS models in this study. As the RCS model takes risk into account and does not skip from one period to the next, it is an ideal tool for determining dose-response associations. In addition, physical activity is painless, which is useful in improving various diseases.

However, the limitations of this study should be acknowledged. First, self-reporting of sleep disorders may be biased as no objective sleep monitoring data were collected. Second, memory bias in the research population’s self-reports of physical activity may have led to outcome bias. Third, the NHANES database is a nutrition-related database, lacking factors such as sedentary behavior [32], the use of sleep-related medication [33], work stress, work hours, and the presence of anxiety and depression tendencies [23, 24], which significantly influence sleep disorders. Additionally, due to the cross-sectional design of this study, causal associations between exercise and sleep disorders cannot be drawn. Overall, this analysis of a representative population from the NHANES database provided good data for clinical research.

A significant negative correlation was observed between physical activity and sleep disturbance. Physical activity showed a significant positive impact on sleep problems, reducing the prevalence of sleep disorders in the general population by 11% with 166.27 MET*h of physical activity per week. In order to facilitate referencing in future research and public health recommendations, these figures were converted from MET*h per week to approximate minutes of Moderate to Vigorous Physical Activity (MVPA), which was defined as ≥ 3 METs [34]. The results indicated a reduction in sleep disorder mortality following a self-reported physical activity at MVPA of 0–7.92 h per day.

## Data Availability

The NHANES dataset is publicly available online, accessible at cdc.gov/nchs/nhanes/index.htm.
